# Patterns of use and perceived value of social media for population health among population health stakeholders: a cross-sectional web-based survey

**DOI:** 10.1186/s12889-021-11370-y

**Published:** 2021-07-05

**Authors:** Sungwon Yoon, Sharon Wee, Vivian S. Y. Lee, Jing Lin, Julian Thumboo

**Affiliations:** 1 Regional Health System, Singapore Health Services, Singapore, Singapore; 2 Programme in Health Services and Systems Research, Duke-NUS Medical School, Singapore, Singapore; 3 Department of Rheumatology and Immunology, Singapore General Hospital, Singapore, Singapore

**Keywords:** Social media, Population health, Cross-sectional survey, Healthcare professionals, Social care professionals

## Abstract

**Background:**

Although existing studies have described patterns of social media use in healthcare, most are focused on health professionals in one discipline. Population health requires a multi-disciplinary approach to ensure diversity and to include diverse stakeholders. To date, what is known about using social media in population health is focused on its potential as a communication tool. This study aims to investigate patterns of use and perceived value of social media usage among stakeholders in population health practice, policy, or research.

**Methods:**

We conducted a web-based survey of delegates attending the Singapore Population Health Conversations and Workshop. We designed a 24-item questionnaire to assess 1) social media use in terms of type of platform and frequency of use; 2) perceptions of social media relevance and impact on population health; and 3) top three areas in population health that would benefit from social media. We used descriptive and logistic regression analyses to assess the relationships between variables.

**Results:**

Of the 308 survey respondents, 97.7% reported that they use social media in some form. Messaging (96.8%) was the most dominant activity when using social media. Challenges in implementing social media for population health were time investment by health care professionals (56.2%) and patient adoption (52.9%). The top three population health areas that would benefit most from using social media were the *promotion of healthy behaviors* (60.7%), *community engagement* (47.7%), and *preventive care* (40.6%). Older respondents (> = 40 years) were less likely to view social media as useful for the *promotion of healthy behaviors* (OR = 0.34; 95% CI: 0.19–0.60). Non-social/healthcare professionals were more likely to consider social media to be useful for *community engagement* (OR = 1.74; 95% CI: 1.10–2.76). For *preventive care*, older respondents (OR = 0.51; 95% CI: 0.32–0.82) and non-social/healthcare professionals were less likely to view social media as useful (OR = 0.61; 95% CI: 0.38–0.97).

**Conclusions:**

Our findings suggest that it may be important to select the specific care areas that would benefit most from using social media. The time investment needed by population health professionals should be fully addressed in planning to maximize the application and potential value of social media.

**Supplementary Information:**

The online version contains supplementary material available at 10.1186/s12889-021-11370-y.

## Background

The growth of social media has changed the way we communicate. The number of social media users worldwide was estimated to be 3.6 billion in 2020 [1, 2]. In many parts of the world, social media is becoming an integral part of our lives. In Singapore, more than 4.6 million people were active social media users as of 2020, representing 79% of the total population [3, 4]. In public health and medicine, social media has been defined as the collective of online communication channels that allow for real-time and on-the-go communication [5]. Social media platforms include wikis (e.g., Wikipedia), messaging (e.g., WhatsApp and Telegram), social networking sites (e.g., Facebook, LinkedIn), media-sharing sites (e.g., YouTube, Instagram, Snapchat), blogs and micro-blogs (e.g., Blogger, Twitter) and immersive worlds (e.g., Second Life) [5, 6].

In healthcare settings, social media has become an increasingly popular tool for communication and information sharing [7]. This trend is demonstrated by the number of systematic reviews published over the past decade: from 2004 to 2009, two systematic reviews were conducted on the topic of social media and health whereas from 2014 to 2016, the number of systematic reviews increased to thirty-four [5]. There are two main reasons why social media tools have been employed in healthcare. The first reason is to improve reach and engagement of patients and the public [8]. To this end, various health departments and agencies have used social media tools to disseminate health information (e.g. in medical emergencies [9] and mass media educational campaigns [10]) and/or to support chronic disease self-management programs [11]. Leading healthcare organizations such as the US Centers for Disease Control and Prevention [12, 13], National Health Service in the United Kingdom [14], and Kaiser Permanente [15] have maintained a significant social media presence, and have actively encouraged social media usage by providing toolkits for their employees to generate content [16, 17]. Interventions tapping on social media for patients with particular health concerns (e.g. Human Immunodeficiency Virus, arthritis) have also been well-received [11, 18].

The second reason why social media tools have been employed in healthcare is for communication among healthcare professionals, e.g. to facilitate exchange of information and for professional networking [8, 19]. Traditionally, professional networking and information sharing have taken place at scientific meetings and conferences. However, social media offers a new avenue for communication for professional development [20–22] and has proven especially useful during the COVID-19 pandemic [23, 24]. Indeed, one study reported that 53 and 35% of healthcare professionals used social media to exchange medical knowledge and enhance their productivity respectively [19]. Dieleman and Duncan [25] described how healthcare professionals utilized an online discussion group to exchange professional advice, establish direct personal contacts among group members, and share materials such as updated policies and protocols. A recent integrative review also found that healthcare professionals used social media to develop virtual communities that facilitated knowledge sharing and evidence-informed practice [26]. Importantly, the review identified a key limitation in the current literature: most published studies have largely focused on professionals in a single homogeneous discipline for a discrete clinical specialty.

Population health is defined as the “health outcomes of a group of individuals” and the larger community [27]. Population health also extends beyond the provision of traditional medical care to encompass social, environmental, economic, and cultural factors which influence health. The nature of population health necessitates a multidisciplinary approach to ensure diversity and to include health and social care professionals [28, 29]. Social media can be a useful avenue to integrate health and social care services [30–32]. An integrative review of social media use by health and social care professionals demonstrated the potential of virtual communication via social media for inter-professional collaboration, but highlighted that active participation from members was required [33]. To date, what is known about using social media in population health is focused on its potential as a communication tool. Little is known about the utility and acceptability of the use of social media for various population health areas as perceived by the stakeholders in population health [34, 35]. Therefore, this study aimed to investigate patterns of use and perceived value of social media usage for population health among stakeholders in population health practice, policy, or research in Singapore.

Singapore is an urban city-state in Southeast Asia, with a rapidly aging population. The proportion of residents aged 65 years and over has increased from 8.7% in 2008 to 14.4% in 2019 [36], and this figure is expected to double to 27% by 2030. Singapore’s national healthcare expenditure has correspondingly increased from $13 billion in 2012 to $22 billion in 2017, or about 11% per annum [37]. To address the challenges of an aging population, the Ministry of Health developed and is implementing a strategic plan for population health which includes community care transformation to better integrate social and healthcare services [38, 39]. Previously, health and social care services were managed separately, with at times suboptimal collaboration among health and social care professionals [40]. Despite care integration efforts, a recent study suggests that there remains a lack of interprofessional communication among health and social care professionals beyond their organizational and professional boundaries [41]. The present paper represents an attempt to gain a better understanding of the receptivity of social media as a potential platform for improving communication and quality community care as perceived by health and social care professionals in population health.

## Methods

### Study design

We designed and conducted a cross-sectional survey using survey software (i.e., SurveyMonkey). The survey was completed anonymously to encourage honest and unbiased responses. Respondents were delegates at the *Singapore Population Health Conversations and Workshop*, an annual nation-wide networking event for practitioners of population health in Singapore that is aimed at sharing experiences and discussing new initiatives in the emerging field of population health [42]. The delegates registered for the workshop with an email address, through which we invited them to participate in the survey by selecting the survey link to participate (www.surveymonkey.com/r/3YDVDHG). Following internationally accepted ethical codes, respondents were duly informed of the purpose of the survey and were reminded of their participation rights before proceeding to take the survey. This study was approved by SingHealth Institutional Review Board (CIRB ref. 2019/2240). We closed the survey link after the workshop ended.

### Questionnaire development

The questionnaire was developed and adapted based on a review of relevant literature [7, 31, 43–46]. It was pretested among ten health and social care professionals and modified according to their feedback. The 24-item questionnaire (Additional File 1) comprised two sections. The first section consisted of questions designed to gather information on respondents’ social media usage in terms of type of platform and frequency of use (daily; weekly; monthly; infrequently/none), social media usage in their organization (yes/no) and perceptions of the relevance and impact of social media on population health (i.e. benefits and limitations of social media for population health work, target population health areas, target age group and target recipients most likely to benefit from using social media). The second section collected information on the respondents’ characteristics such as age, sex, and primary role in population health. The survey was conducted in English (which was the language used for the host event) and required approximately 15 min to complete.

### Classification of variables

The type of social media platform was categorized into 4 functional groups: messaging (WhatsApp and Telegram), networking (Facebook and LinkedIn), media sharing (YouTube, Instagram, and Snapchat), and microblogging (Twitter) [6, 47]. The frequency of social media usage by type of social media platform was dichotomized into frequent use (daily, weekly, monthly) and infrequent use (infrequently/none). Organizational use of social media was dichotomized into yes (selected area(s) in which organization used social media) and no (none selected). Level of understanding of population health was categorized into 4 groups based on a rating of 1 to 10: 1–4 (not at all/ a little); 5–6 (moderately good); 7–8 (good); and 9–10 (very good).

### Statistical analysis

Descriptive summary statistics were presented as counts and percentages. Responses from the following questions were used for model building:

- *What are the top three areas in which social media would be useful for population health?*
- *Do you use social media for population health work?*
- *Which social media platform do you use and how frequently do you use it?*
- *In which area(s) does your organization currently use social media for population health?*
- *Please rate your level of understanding pertaining to population health.*

The top three areas in which social media would be useful for population health were the dependent variables. We analyzed factors predicting targeted population health areas that would benefit from using social media using logistic regression analysis.

The baseline models first estimated each of the top three population health areas reported useful by respondents after adjusting for demographic characteristics (i.e., age, sex), and primary role in population health. Subsequently, we added personal use/non-use of social media, frequency of social media usage by type of social media platform, organizational use/non-use of social media for population health work*,* and level of understanding of population health*.*

To improve model fit, we performed a stepwise backward elimination procedure, with a *p*-value more than or equal to 0.1 as the significance level for variable removal. At every step, age, sex, and primary role were kept in the model. We specified the odds ratios, 95% confidence intervals (95% CI), *p*-values, and goodness-of-fit statistics in Additional File 2, and presented significant variables of the final model in the results section. All statistical analyses were conducted using STATA version 15. A *p*-value of less than .05 was considered statistically significant.

## Results

We sent an invitation email and one reminder to all workshop registrants (*n* = 917). Of 412 respondents, 308 completed the survey, yielding a response rate of 33.6%. Only completed surveys were included in the analysis. Characteristics of the respondents are shown in Table 1. Of the respondents, 48.7% were below the age of 40 years and 77.6% were female. Less than half (44.5%) of the respondents were health and social care professionals while the remaining respondents were non-care professionals (e.g., program managers, administrators, researchers, and students). While the vast majority of respondents (97.7%) reported that they used social media in some form, only 22.4% reported that they had used social media for population health work. However, 77.3% reported that their organizations used social media for population health. More than two-thirds of the respondents indicated that their level of understanding of population health was good or moderately good (72.4%).

**Table 1:** Characteristics of respondents

	Overall (%) (***N*** = 308)	
**Age**		
below 40	150	(48.70)
40 and above	158	(51.30)
**Sex**		
Male	69	(22.40)
Female	239	(77.60)
**Primary Role**		
Healthcare professionals and social care professionals	137	(44.48)
Others (program managers, researchers, administrators)	169	(54.87)
**Use of Social Media**		
Yes	301	(97.73)
No	7	(2.27)
**Use of Social Media for Population Health work**		
Yes	69	(22.40)
No	239	(77.60)
**Organisational use of Social Media for Population Health**		
Yes	238	(77.27)
No	70	(22.73)
**Level of Understanding of Population Health**		
Not at all/A little	69	(22.40)
Moderately good	125	(40.58)
Good	98	(31.82)
Very good	16	(5.19)

**Fig. 1:**
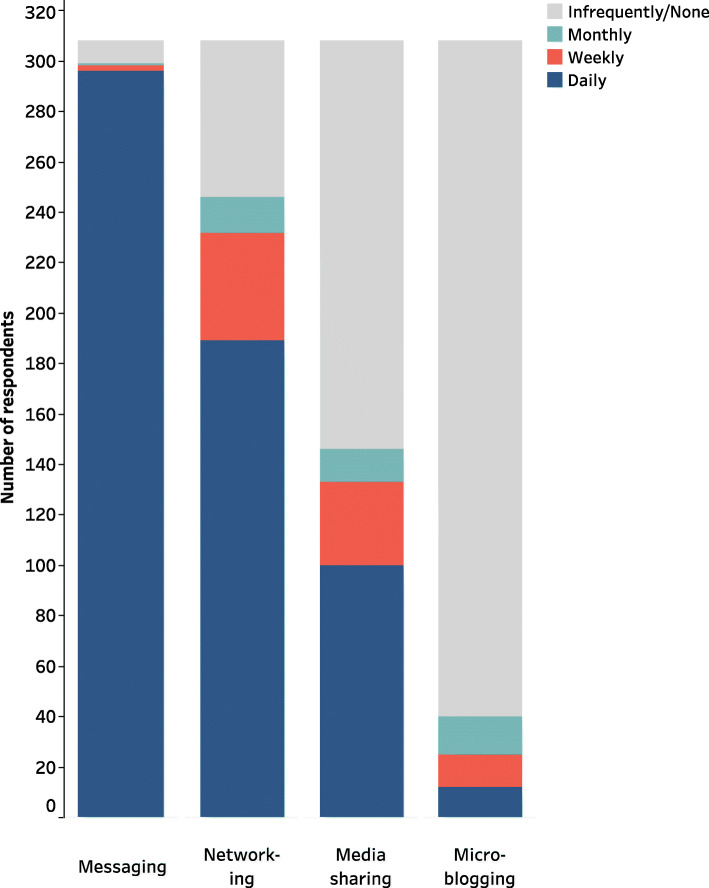
Frequency of social media use by type of social media platform

Figure 1 shows the frequency of social media use by type of social media platform. Messaging (i.e., WhatsApp and Telegram) was the most dominant activity when using social media, with 96.1% of respondents using it daily, followed by networking (i.e., Facebook and LinkedIn; 61.4% used these daily). Media sharing (i.e., YouTube, Instagram, and Snapchat) and microblogging (i.e., Twitter) were the two least frequently used social media platforms (32.5 and 3.9% used these daily respectively).

Perceptions of the utility of social media for population health are presented in Table 2. Respondents were asked to choose three areas in population health that would benefit most from using social media. The top three areas identified were the *promotion of healthy behaviors* (60.7%), *community engagement* (47.7%), and *preventive care* (40.6%). The most commonly reported target age group was 30–39 years (92.2%) followed by 40–49 years (89.6%). Overall, respondents’ (87.3%) views were that caregivers would benefit most from using social media as a communication tool for population health followed by healthcare professionals in the community (84.1%). Approximately three quarters agreed that social media sites or apps (77.0%) and face-to-face group sessions (73.1%) were two useful modes of communication for population health work. The least useful mode of communication for population health work was telephone calls (27.3%) followed by text messages (33.1%). The main challenges in implementing social media for population health were time investment by health care professionals (56.2%) and patient adoption (53.0%).

**Table 2:** Perceptions of social media use for population health

	n	(%)^a^
**Population health areas that would benefit from social media use**		
Promotion of health behaviors	187	(60.71)
Community engagement	147	(47.73)
Preventive care (e.g. diabetes screening, immunization)	125	(40.58)
Chronic disease management	119	(38.64)
Care coordination	78	(25.32)
Mental health	55	(17.86)
Social support	54	(17.53)
Health-social interface	56	(18.18)
Population health policy	34	(11.04)
Intermediate to long term care	22	(7.14)
Palliative/End-of-life care	18	(5.84)
Research	10	(3.25)
Acute disease management	9	(2.92)
Post-acute care recovery	8	(2.60)
**Age groups (years) that would benefit from social media use**		
12 and below	46	(14.94)
13–19	160	(51.95)
20–29	256	(83.12)
30–39	284	(92.21)
40–49	276	(89.61)
50–59	210	(68.18)
60–69	117	(37.99)
70 and above	56	(18.18)
**Recipients that would benefit from social media use**		
Caregivers	269	(87.34)
Healthcare professionals in the community	259	(84.09)
Patients	238	(77.27)
Healthcare professionals in acute hospitals	170	(55.19)
Social care professionals in acute hospitals	161	(52.27)
**Useful modes of communication in population health**		
Social media sites/apps	237	(76.95)
Face-to-face group sessions	225	(73.05)
Websites	165	(53.57)
Text messages	102	(33.12)
Telephone calls	84	(27.27)
**Main challenges for the use of social media**		
Time investment by health care professionals	173	(56.17)
Patient adoption	163	(52.92)
Infrastructure development of technological approaches	158	(51.30)
Providing supervision and follow-up	151	(49.03)
Adoption by health care professionals	140	(45.45)
Cost investment	133	(43.18)

^a^Total number does not add up to 308 because multiple selections were allowed

Table 3 presents the associations between respondent characteristics and the top three population health areas reported useful by respondents. Older respondents were less likely to view social media as useful for the *promotion of healthy behaviors* (OR = 0.34; 95% CI: 0.19–0.60; *P* < .001). Non-health/social care professionals were more likely to consider social media as useful for *community engagement* (OR = 1.74; 95% CI: 1.10–2.76; *P* < .05). Lastly, older respondents and non-health/social care professionals were less likely to consider social media to be useful for *preventive care* (OR = 0.51; 95% CI: 0.32–0.82; *P* < .05; OR = 0.61; 95% CI: 0.38–0.97; *P* < .05) while female respondents were more likely to view social media as useful (OR = 1.81; 95% CI: 1.01–3.24; *P* < .05).

**Table 3:** Association between participant characteristics and target population health areas

	Promotion of healthy behaviors			Community Engagement			Preventive Care		
	OR	[95% CI]	***p***-value	OR	[95% CI]	***p***-value	OR	[95% CI]	***p***-value
**Age**									
below 40	*Ref*			*Ref*			*Ref*		
40 and above	0.34	[0.19–0.60]	< 0.001	1.24	[0.78–1.95]	0.36	0.51	[0.32–0.82]	< 0.05
**Sex**									
Male	*Ref*			*Ref*			*Ref*		
Female	0.85	[0.48–1.51]	0.58	0.81	[0.46–1.40]	0.45	1.81	[1.01–3.24]	< 0.05
**Primary role**									
Health and social care professionals	*Ref*			*Ref*			*Ref*		
Others (program managers, researchers, administrators)	1.15	[0.72–1.84]	0.57	1.74	[1.10–2.76]	< 0.05	0.61	[0.38–0.97]	< 0.05

## Discussion

This study investigated the patterns of social media use and perceived value of social media for population health among health, social care and other professionals working in population health. To the best of our knowledge, this is the first study to examine specific areas in which population health would benefit from using social media, from the perspective of population health stakeholders.

The vast majority of respondents were regular social media users, which is similar to prior studies [48, 49]. Social media was used predominantly for messaging, followed by networking and media sharing. Microblogging was the least frequently used social media platform. This finding indicates that social media may have a potential to provide a medium for communication and networking for professionals in population health. This finding is in line with other studies that found healthcare professionals often used a communication application, such as WhatsApp or Facebook [19]. Research shows that interaction and communication with a diverse range of professionals beyond an individual organization or a single specialty enabled healthcare workers to make more informed decisions and to develop work-related resources [26]. Future implementation should consider how existing social media platforms for communication and networking could be usefully integrated into the development of shared spaces for population health professionals to interact.

We found that most respondents viewed social media as a useful mode of communication for population health, particularly for caregivers. This perspective is supported by studies showing that online support delivered via social media decreased the burden of care for informal caregivers and increased the contact time clinicians had with patients and their caregivers [50]. A scoping review of 284 articles also found that the use of social media improved self-care and clinical decision-making for patients and their caregivers [51]. Similarly, caregivers reported decreased burden, reduced stress, and increased support following a 6-week intervention using online peer support groups [52]. Despite these positive outcomes, a study in Singapore showed that an online forum was not well received by caregivers of patients with advanced cancer, citing reasons such as not being computer savvy, rarely surfing the internet and not feeling comfortable sharing information with strangers on an online platform [53]. These findings, together with the finding from our study, indicate that digital literacy, issues of trust and privacy, and sufficient resources are needed for successful caregiver adoption of social media.

Our study found social media to be of most value for the following three areas in population health: i) promotion of healthy behaviors, ii) community engagement, and iii) preventive care. The younger respondents were, the more likely they were to view social media as useful for the *promotion of healthy behaviors* and *preventive care*. Younger respondents would have grown up with digital technology and would therefore be more familiar with social media campaigns promoting healthy behaviors and encouraging preventive care. In contrast, older respondents may not be active users of social media, and hence were less likely to come across population health campaigns through social media platforms. The overall positive perceptions of social media in younger age groups is similar to the findings of other studies that younger health care workers [49] and doctors [48] were more likely to support the use of social media for healthcare-related tasks than their older counterparts.

While health and social care professionals viewed social media as useful for *preventive care*, they did not view it to be useful for *community engagement*. This finding could be explained by the nature of population health work. Health and social care professionals are directly involved in the care of the population and therefore they are cognizant of the effort required for community engagement. Community engagement involves empowering the local community with skills to take ownership and make decisions that improve the health and wellbeing of residents [54]. This entails fostering collaboration and trust through relationship building with community members [41, 55, 56]. As relationships are built through in-person interactions which involve face-to-face contact, health and social care professionals responses might reflect the view that social media may not replace in-person communication when building and establishing relationships in the local community [56]. This also echoes our survey finding that face-to-face sessions were perceived to be the second most useful mode of communication (followed by social media sites and applications).

Overall, our findings show that when implementing social media for population and community care initiatives, it may be important to select the specific care areas that would benefit most from using social media. For example, health promotion messages and campaigns can be usefully integrated into social media platforms to influence the behaviors of intended audiences; however face-to-face contact might be selected for activities that require and / or aim to build professional-patient relationships. In addition, it may be important to take into account the age of population health professionals when implementing population health interventions that harness social media. Lastly, notwithstanding the perceived benefits of social media for population health, it is not without drawbacks. The time investment needed by population health professionals and patient adoption should be addressed when developing social media programs for population health [57–59].

While focusing on population health areas in which social media might be beneficial, attention could also be given to counter misleading health information disseminated through social media that may have deleterious effects on population health. A recent systematic review observed an increased prevalence of misinformation on social media primarily related to vaccines and infectious diseases [60]. The negative public health effects of misinformation through social media have been especially pertinent in light of the ongoing COVID-19 pandemic and vaccine hesitancy [61]. Future research should examine how best to combat the negative effects of health-related misinformation on social media.

The strength of this study included the participation of population health practitioners from multiple disciplines, which provided broad perspectives of the utility of social media from different stakeholders. Our findings should also be interpreted in light of the following limitations: the survey was based on opportunistic sampling and participation was voluntary. First, the relatively low response rate (33.6%) might have resulted in potential biases limiting generalizability of our findings. Second, in our study, only 2.3% of respondents reported that they did not use social media. This is relatively low compared to surveys conducted elsewhere [62] and may reflect selection bias, where only those familiar with social media chose to respond to the survey. Third, this study is cross-sectional and therefore causal inferences cannot be drawn. Lastly, our study did not include some important population health stakeholders, in particular patients and community members. Future studies should examine the perceptions of these stakeholders on the value of social media for population health.

## Conclusions

This study provides valuable insights into the views of population health stakeholders on the value of social media for population health work, and can inform the design of population health programs and interventions that harness social media platforms. Optimizing population health and community care requires a greater attention to enhancing person-centered care and health-social interface. While social media can be a useful tool for population health, understanding target audiences, specific care areas, and needs would improve its optimal adoption and utilization.

## Supplementary Information

**Additional file 1:** Word format of the questionnaire that was uploaded online.

**Additional file 2:** Supplementary Tables showing the logistic regression models used for the stepwise backward elimination procedure.

## Data Availability

The dataset used and analyzed during the current study is available from the corresponding author on reasonable request.

## Abbreviations

CDC: Centers for Disease Control and Prevention
NHS: National Health Service
CI: Confidence interval
OR: Odds ratio
