# Supplementary material for: Patterns of use and perceived value of social media for population health among population health stakeholders: a cross-sectional web-based survey
Source: BMC Public Health. 2021 Jul 5;21:1312. doi: 10.1186/s12889-021-11370-y (PMC8256205; doi:10.1186/s12889-021-11370-y)
Supplement: Supplementary file 1 — Additional file 1: Word format of the questionnaire that was uploaded online. [file 12889_2021_11370_MOESM1_ESM.docx]

*Thank you for registering to our workshop. We would like to invite you to take about 10-15 minutes to participate in this short anonymous survey. Your participation is voluntary and you may stop at any time. Please be assured that no identifiable data would be collected in this survey, which has been approved by the SingHealth Institutional Review Board.*

# Questionnaire

***Population Health Workshop and Conversation 2019***

*Section A. The use of social media is growing rapidly, and we would like your thoughts on how it can be used in population health. As members and contributors to the health system, your input is vital in helping us design initiatives that are relevant and impactful.* ***Social media*** *refers to websites and internet applications that allow users to create and share content or to participate in social networking.* ***Population health*** *can be defined as an approach that focuses on personal, behavioural and environmental determinants of health that influence the health outcomes of populations.*

1. **Do you use social media?**

- Yes (Proceed to Q3)
- No

**What is the reason for not using social media? (select all that apply)**

- Lack of knowledge on how to use social media
- Not suitable for my needs
- Safety and privacy concerns
- Validity of information
- Not interested
- No time
- Others; please specify ___________________________________

(Proceed to Q6)

**Which social media platform do you use and how frequently do you use it? (select all that apply)**

|  | **Social Media Platform** | **Daily** | **Weekly** | **Monthly** | **Infrequently** |
| --- | --- | --- | --- | --- | --- |
|  | Facebook |  |  |  |  |
|  | Whatsapp |  |  |  |  |
|  | LinkedIn |  |  |  |  |
|  | Telegram |  |  |  |  |
|  | Instagram |  |  |  |  |
|  | Twitter |  |  |  |  |
|  | Snapchat |  |  |  |  |
|  | Others; please specify __________________ |  |  |  |  |
|  | For staff of SingHealth Institutions | | |  |  |
|  | Facebook @ Workplace |  |  |  |  |
|  | Tiger Text |  |  |  |  |

1. **Do you use social media for population health work?**

- Yes
- No (proceed to Q6)

**Which social media platform do you use for population health work and how frequently do you use it? (select all that apply)**

|  | **Social Media Platform** | **Daily** | **Weekly** | **Monthly** | **Infrequently** |
| --- | --- | --- | --- | --- | --- |
|  | Facebook |  |  |  |  |
|  | Whatsapp |  |  |  |  |
|  | LinkedIn |  |  |  |  |
|  | Telegram |  |  |  |  |
|  | Instagram |  |  |  |  |
|  | Twitter |  |  |  |  |
|  | Snapchat |  |  |  |  |
|  | Others; please specify __________________ |  |  |  |  |
|  | For staff of SingHealth Institutions staff | | |  |  |
|  | Facebook @ Workplace |  |  |  |  |
|  | Tiger Text |  |  |  |  |

1. **If we are to harness social media as communication tools for population health, which of the following group(s) would most likely benefit? (select all that apply)**
   - Patients
   - Caregivers
   - Healthcare providers in the community
   - Healthcare providers in acute hospital
   - Social service providers in the community
   - Social service providers in acute hospital
   - Others; please specify _________________________________
2. **If we are to harness social media as communication tools for population health, which age group(s) would best be targeted? (select all that apply)**
   - 12 years and below
   - 13 – 19 years
   - 20 – 29 years
   - 30 – 39 years
   - 40 – 49 years
   - 50 – 59 years
   - 60 – 69 years
   - 70 years and above
3. **What are the most useful modes of communication for population health work? (select all that apply)**
   - Face-to-face group sessions
   - Social media sites/ apps
   - Text messages
   - Websites
   - Telephone calls
   - Others; please specify _________________________________
4. **What are the top 3 areas in which social media would be useful for population health?**
   - Chronic disease management
   - Promotion of healthy behaviours/ Wellness
   - Care Coordination
   - Health-social interface
   - Social support
   - Preventive care (e.g. diabetes screening, immunization)
   - Mental Health
   - Post-acute care recovery
   - Intermediate to long term care
   - Palliative care or end-of-life care
   - Acute disease management

Community engagement

Population health policy

Research

Others (please specify ___________________________)

1. **Please rank your top 3 choices with 1 being the most useful, 2 being the second most useful and 3 being the third most useful.**
2. **In which area(s) does your organisation currently use social media for population health? (select all that apply)**
   - Chronic disease management
   - Promotion of healthy behaviours/ Wellness
   - Care Coordination
   - Health-social interface
   - Social support
   - Preventive care (e.g. diabetes screening, immunization)
   - Mental Health
   - Post-acute care recovery
   - Intermediate to long term care
   - Palliative care or end-of-life care
   - Acute disease management

Community engagement

Population health policy

Research

None

Others (please specify _______________________________)

1. **What would be the main challenges in scaling social media for population health? (select all that apply)**
   - Time investment by health care providers
   - Adoption by health care providers
   - Patient adoption
   - Infrastructure development of technological approaches
   - Providing supervision and follow-up
   - Cost investment
   - Others; please specify _______________________________________

*Section B. We would like to ask about yourself and your experience to help us understand your responses better.*

1. **Please rate your level of understanding pertaining to population health, on a scale of 1-10, with 1 being ‘**I know nothing about population health’, and 10 being ‘I live and breathe population health”.

| **1** |  | **2** |  | **3** |  | **4** |  | **5** |  | **6** |  | **7** |  | **8** |  | **9** |  | **10** |
| --- | --- | --- | --- | --- | --- | --- | --- | --- | --- | --- | --- | --- | --- | --- | --- | --- | --- | --- |
|  |  |  |  |  |  |  |  |  |  |  |  |  |  |  |  |  |  |  |

1. **Are you involved in population health work** (e.g. care delivery and management, prevention, wellness, health promotion efforts, community engagement and resources, population health policy and research)**?**
   - **Yes**
   - **No**
   - **Not sure**
2. **Please rank the following areas of population health; 1 being the most important and 6 the least important.**

| Rank | Population Health Area |
| --- | --- |
|  | Empower people/ patients to take charge of their health |
|  | Improve care transition and management through relationship building and communication |
|  | Enhance health-social care interface |
|  | Improve respite care services for long-term caregivers |
|  | Develop primary care as a driving force for care integration |
|  | Capacity building for service providers |

**Do you have additional comments on the priority areas? (optional)**

**________________________________________________________________________**

**Are there other areas of population health priority not listed above? (optional)**

**________________________________________________________________________**

**Which one of the following categories best describes your primary role?**

Senior Management/ Director

Administrator

Clerical/ support staff

Educator/Trainer

Doctor

Nurse

Allied health professional

Social service provider

IT professional

Patient

Caregiver

Academic/ Researcher/ Analyst

Student

Other, please specify_______________________________

**You would consider yourself predominantly as a:**

Health care provider

Social care provider

Not applicable

**What type of organization do you work for?**

Healthcare institution (e.g. hospital, medical centre, clinic)

Government agency (e.g. MOH, MSF, HPB etc)

College or University

Research Institute (e.g. SCRI, SERI)

Private industry or business

Voluntary Welfare Organisation

Social Service Organisation (e.g. SAC, FSC)

Grassroots organisation (e.g. RC, CDC)

Foundation/ Society

Other, please specify __________________________________

**How long have you worked in the field of population health?** ______ year(s) [if less than a year, put ‘0’]

I do not work in population health

**How long have you been at your current position?** _________ year(s) [if less than a year, put ‘0’]

**What is your gender?**

Male

Female

**What is your age?**

Below 20 years

20-29

30-39

40-49

50-59

60-69

70 years or over
