# Supplementary material for: Patterns of use and perceived value of social media for population health among population health stakeholders: a cross-sectional web-based survey
Source: BMC Public Health. 2021 Jul 5;21:1312. doi: 10.1186/s12889-021-11370-y (PMC8256205; doi:10.1186/s12889-021-11370-y)
Supplement: Supplementary file 2 — Additional file 2: Supplementary Tables showing the logistic regression models used for the stepwise backward elimination procedure. [file 12889_2021_11370_MOESM2_ESM.docx]

**Appendix 1. Logistic regression of the reported usefulness of social media for promotion of healthy behaviors or wellness on, demographic characteristics, personal use/non-use of social media, frequency of social media usage by types of social media platforms, organizational use/non-use of social media for population health work, and level of understanding of population health.**

|  | **Baseline model** | | | | **Model 1** | | | | **Model 2** | | | |
| --- | --- | --- | --- | --- | --- | --- | --- | --- | --- | --- | --- | --- |
|  | **OR** | **[95% CI]** | | **p-value** | **OR** | **[95% CI]** | | **p-value** | **OR** | **[95% CI]** | | **p-value** |
| **Age** |  |  |  |  |  |  |  |  |  |  |  |  |
| below 40 | *Ref* |  |  |  | *Ref* |  |  |  | *Ref* |  |  |  |
| 40 and above | 0.44 | [0.28 - | 0.71] | < 0.05 | 0.35 | [0.20 - | 0.63] | < 0.001 | 0.34 | [0.19 - | 0.60] | < 0.001 |
|  |  |  |  |  |  |  |  |  |  |  |  |  |
| **Sex** |  |  |  |  |  |  |  |  |  |  |  |  |
| Male | *Ref* |  |  |  | *Ref* |  |  |  | *Ref* |  |  |  |
| Female | 0.81 | [0.46 - | 1.43] | 0.46 | 0.87 | [0.48 - | 1.56] | 0.63 | 0.85 | [0.48 - | 1.51] | 0.58 |
|  |  |  |  |  |  |  |  |  |  |  |  |  |
| **Primary role** |  |  |  |  |  |  |  |  |  |  |  |  |
| Healthcare professionals and social care professionals | *Ref* |  |  |  | *Ref* |  |  |  | *Ref* |  |  |  |
| Others | 1.13 | [0.71 - | 1.81] | 0.61 | 1.08 | [0.66 - | 1.76] | 0.76 | 1.15 | [0.72 - | 1.84] | 0.57 |
|  |  |  |  |  |  |  |  |  |  |  |  |  |
| **Use of Social Media for Population Health work** |  |  |  |  |  |  |  |  |  |  |  |  |
| No | - | - |  | - | *Ref* |  |  |  | - | - |  | - |
| Yes | - | - |  | - | 0.74 | [0.41 - | 1.33] | 0.31 | - | - |  | - |
|  |  |  |  |  |  |  |  |  |  |  |  |  |
| **Organizational use of Social Media for Population Health** |  |  |  |  |  |  |  |  |  |  |  |  |
| No | - | - |  | - | *Ref* |  |  |  | - | - |  | - |
| Yes | - | - |  | - | 0.87 | [0.49 - | 1.56] | 0.64 | - | - |  | - |
|  |  |  |  |  |  |  |  |  |  |  |  |  |
| **Level of Understanding of Population Health** |  |  |  |  |  |  |  |  |  |  |  |  |
| Not at all/A little | - | - |  | - | *Ref* |  |  |  | - | - |  | - |
| Moderately good | - | - |  | - | 0.76 | [0.40 - | 1.44] | 0.40 | - | - |  | - |
| Good | - | - |  | - | 0.88 | [0.45 - | 1.72] | 0.71 | - | - |  | - |
| Very good | - | - |  | - | 1.29 | [0.38 - | 4.39] | 0.68 | - | - |  | - |
|  |  |  |  |  |  |  |  |  |  |  |  |  |
| **Frequency of social media usage** |  |  |  |  |  |  |  |  |  |  |  |  |
| **Networking** |  |  |  |  |  |  |  |  |  |  |  |  |
| Infrequently | - | - |  | - | *Ref* |  |  |  | - | - |  | - |
| Frequently | - | - |  | - | 1.35 | [0.75 - | 2.43] | 0.32 | - | - |  | - |
| **Messaging** |  |  |  |  |  |  |  |  |  |  |  |  |
| Infrequently | - | - |  | - | *Ref* |  |  |  | - | - |  | - |
| Frequently | - | - |  | - | 0.89 | [0.22 - | 3.61] | 0.87 | - | - |  | - |
| **Media sharing** |  |  |  |  |  |  |  |  |  |  |  |  |
| Infrequently | - | - |  | - | *Ref* |  |  |  | *Ref* |  |  |  |
| Frequently | - | - |  | - | 0.60 | [0.33 - | 1.11] | 0.11 | 0.62 | [0.35 - | 1.09] | 0.10 |
| **Microblogging** |  |  |  |  |  |  |  |  |  |  |  |  |
| Infrequently | - | - |  | - | *Ref* |  |  |  | - | - |  | - |
| Frequently | - | - |  | - | 0.93 | [0.38 - | 2.29] | 0.88 | - | - |  | - |
|  |  |  |  |  |  |  |  |  |  |  |  |  |
| *Baseline model: Age, sex and primary role in health system. Model fit statistics are, X² (3, N=584) = 13.10, p < .05, AIC = 407.62.* | | | | | | | | | | | | |
| *Model 1: Baseline model, personal use/non-use of social media, frequency of social media usage by types of social media platforms, organizational use/non-use of social media for population health work, and level of understanding of population health. Model fit statistics are X² (12, N=584) = 19.60, p = 0.08, AIC = 419.13.* | | | | | | | | | | | | |
| *Model 2: Backward elimination was performed on Model 1 to obtain the final model. Model fit statistics are, X² (4, N=584) = 15.96, p < .05, AIC = 406.77.* | | | | | | | | | | | | |

**Appendix 2. Logistic regression of the reported usefulness of social media for community engagement on, demographic characteristics, personal use/non-use of social media, frequency of social media usage by types of social media platforms, organizational use/non-use of social media for population health work, and level of understanding of population health.**

|  | **Baseline model** | | | | **Model 1** | | | | **Model 2** | | | |
| --- | --- | --- | --- | --- | --- | --- | --- | --- | --- | --- | --- | --- |
|  | **OR** | **[95% CI]** | | **p-value** | **OR** | **[95% CI]** | | **p-value** | **OR** | **[95% CI]** | | **p-value** |
| **Age** |  |  |  |  |  |  |  |  |  |  |  |  |
| below 40 | *Ref* |  |  |  | *Ref* |  |  |  | *Ref* |  |  |  |
| 40 and above | 1.19 | [0.76 - | 1.87] | 0.45 | 1.18 | [0.69 - | 2.04] | 0.55 | 1.24 | [0.78 - | 1.95] | 0.36 |
|  |  |  |  |  |  |  |  |  |  |  |  |  |
| **Sex** |  |  |  |  |  |  |  |  |  |  |  |  |
| Male | *Ref* |  |  |  | *Ref* |  |  |  | *Ref* |  |  |  |
| Female | 0.87 | [0.51 - | 1.49] | 0.61 | 0.81 | [0.46 - | 1.43] | 0.47 | 0.81 | [0.46 - | 1.40] | 0.45 |
|  |  |  |  |  |  |  |  |  |  |  |  |  |
| **Primary role** |  |  |  |  |  |  |  |  |  |  |  |  |
| Healthcare professionals and social care professionals | *Ref* |  |  |  | *Ref* |  |  |  | *Ref* |  |  |  |
| Others | 1.66 | [1.05 - | 2.61] | < 0.05 | 1.75 | [1.09 - | 2.82] | < 0.05 | 1.74 | [1.10 - | 2.76] | < 0.05 |
|  |  |  |  |  |  |  |  |  |  |  |  |  |
| **Use of Social Media for Population Health work** |  |  |  |  |  |  |  |  |  |  |  |  |
| No | - | - |  | - | *Ref* |  |  |  | - | - |  | - |
| Yes | - | - |  | - | 1.06 | [0.60 - | 1.88] | 0.84 | - | - |  | - |
|  |  |  |  |  |  |  |  |  |  |  |  |  |
| **Organizational use of Social Media for Population Health** |  |  |  |  |  |  |  |  |  |  |  |  |
| No | - | - |  | - | *Ref* |  |  |  | - | - |  | - |
| Yes | - | - |  | - | 0.99 | [0.56 - | 1.72] | 0.96 | - | - |  | - |
|  |  |  |  |  |  |  |  |  |  |  |  |  |
| **Level of Understanding of Population Health** |  |  |  |  |  |  |  |  |  |  |  |  |
| Not at all/A little | - | - |  | - | *Ref* |  |  |  | - | - |  | - |
| Moderately good | - | - |  | - | 1.57 | [0.85 - | 2.91] | 0.15 | - | - |  | - |
| Good | - | - |  | - | 1.52 | [0.80 - | 2.91] | 0.21 | - | - |  | - |
| Very good | - | - |  | - | 1.77 | [0.57 - | 5.50] | 0.32 | - | - |  | - |
|  |  |  |  |  |  |  |  |  |  |  |  |  |
| **Frequency of social media usage** |  |  |  |  |  |  |  |  |  |  |  |  |
| **Networking** |  |  |  |  |  |  |  |  |  |  |  |  |
| Infrequently | - | - |  | - | *Ref* |  |  |  | - | - |  | - |
| Frequently | - | - |  | - | 0.89 | [0.50 - | 1.57] | 0.68 | - | - |  | - |
| **Messaging** |  |  |  |  |  |  |  |  |  |  |  |  |
| Infrequently | - | - |  | - | *Ref* |  |  |  | *Ref* |  |  |  |
| Frequently | - | - |  | - | 5.40 | [1.06 - | 27.55] | < 0.05 | 4.90 | [1.00 - | 24.04] | 0.05 |
| **Media sharing** |  |  |  |  |  |  |  |  |  |  |  |  |
| Infrequently | - | - |  | - | *Ref* |  |  |  | - | - |  | - |
| Frequently | - | - |  | - | 0.94 | [0.53 - | 1.66] | 0.82 | - | - |  | - |
| **Microblogging** |  |  |  |  |  |  |  |  |  |  |  |  |
| Infrequently | - | - |  | - | *Ref* |  |  |  | - | - |  | - |
| Frequently | - | - |  | - | 1.29 | [0.54 - | 3.08] | 0.56 | - | - |  | - |
|  |  |  |  |  |  |  |  |  |  |  |  |  |
| *Baseline model: Age, sex and primary role in health system. Model fit statistics are, X² (3, N=584) = 5.45, p = 0.14, AIC = 428.89.* | | | | | | | | | | | | |
| *Model 1: Baseline model, personal use/non-use of social media, frequency of social media usage by types of social media platforms, organizational use/non-use of social media for population health work, and level of understanding of population health. Model fit statistics are X² (12, N=584) = 13.51, p = 0.33, AIC = 438.84.* | | | | | | | | | | | | |
| *Model 2: Backward elimination was performed on Model 1 to obtain the final model. Model fit statistics are, X² (4, N=584) = 10.26, p < .05, AIC = 426.08.* | | | | | | | | | | | | |

**Appendix 3. Logistic regression of the reported usefulness of social media for preventive care on, demographic characteristics, personal use/non-use of social media, frequency of social media usage by types of social media platforms, organizational use/non-use of social media for population health work, and level of understanding of population health.**

|  | **Baseline model** | | | | **Model 1** | | | | **Model 2** | | | |
| --- | --- | --- | --- | --- | --- | --- | --- | --- | --- | --- | --- | --- |
|  | **OR** | **[95% CI]** | | **p-value** | **OR** | **[95% CI]** | | **p-value** | **OR** | **[95% CI]** | | **p-value** |
| **Age** |  |  |  |  |  |  |  |  |  |  |  |  |
| below 40 | *Ref* |  |  |  | *Ref* |  |  |  | *Ref* |  |  |  |
| 40 and above | 0.51 | [0.32 - | 0.82] | < 0.05 | 0.49 | [0.28 - | 0.86] | < 0.05 | 0.51 | [0.32 - | 0.82] | < 0.05 |
|  |  |  |  |  |  |  |  |  |  |  |  |  |
| **Sex** |  |  |  |  |  |  |  |  |  |  |  |  |
| Male | *Ref* |  |  |  | *Ref* |  |  |  | *Ref* |  |  |  |
| Female | 1.81 | [1.01 - | 3.24] | < 0.05 | 1.83 | [1.00 - | 3.33] | 0.05 | 1.81 | [1.01 - | 3.24] | < 0.05 |
|  |  |  |  |  |  |  |  |  |  |  |  |  |
| **Primary role** |  |  |  |  |  |  |  |  |  |  |  |  |
| Healthcare professionals and social care professionals | *Ref* |  |  |  | *Ref* |  |  |  | *Ref* |  |  |  |
| Others | 0.61 | [0.38 - | 0.97] | < 0.05 | 0.58 | [0.35 - | 0.94] | < 0.05 | 0.61 | [0.38 - | 0.97] | < 0.05 |
|  |  |  |  |  |  |  |  |  |  |  |  |  |
| **Use of Social Media for Population Health work** |  |  |  |  |  |  |  |  |  |  |  |  |
| No | - | - |  | - | *Ref* |  |  |  | - | - |  | - |
| Yes | - | - |  | - | 0.76 | [0.42 - | 1.38] | 0.37 | - | - |  | - |
|  |  |  |  |  |  |  |  |  |  |  |  |  |
| **Organizational use of Social Media for Population Health** |  |  |  |  |  |  |  |  |  |  |  |  |
| No | - | - |  | - | *Ref* |  |  |  | - | - |  | - |
| Yes | - | - |  | - | 0.86 | [0.49 - | 1.51] | 0.61 | - | - |  | - |
|  |  |  |  |  |  |  |  |  |  |  |  |  |
| **Level of Understanding of Population Health** |  |  |  |  |  |  |  |  |  |  |  |  |
| Not at all/A little | - | - |  | - | *Ref* |  |  |  | - | - |  | - |
| Moderately good | - | - |  | - | 1.31 | [0.70 - | 2.45] | 0.40 | - | - |  | - |
| Good | - | - |  | - | 1.37 | [0.70 - | 2.68] | 0.36 | - | - |  | - |
| Very good | - | - |  | - | 1.21 | [0.37 - | 3.92] | 0.75 | - | - |  | - |
|  |  |  |  |  |  |  |  |  |  |  |  |  |
| **Frequency of social media usage** |  |  |  |  |  |  |  |  |  |  |  |  |
| **Networking** |  |  |  |  |  |  |  |  |  |  |  |  |
| Infrequently | - | - |  | - | *Ref* |  |  |  | - | - |  | - |
| Frequently | - | - |  | - | 1.26 | [0.69 - | 2.29] | 0.45 | - | - |  | - |
| **Messaging** |  |  |  |  |  |  |  |  |  |  |  |  |
| Infrequently | - | - |  | - | *Ref* |  |  |  | - | - |  | - |
| Frequently | - | - |  | - | 1.05 | [0.24 - | 4.53] | 0.95 | - | - |  | - |
| **Media sharing** |  |  |  |  |  |  |  |  |  |  |  |  |
| Infrequently | - | - |  | - | *Ref* |  |  |  | - | - |  | - |
| Frequently | - | - |  | - | 0.90 | [0.50 - | 1.63] | 0.74 | - | - |  | - |
| **Microblogging** |  |  |  |  |  |  |  |  |  |  |  |  |
| Infrequently | - | - |  | - | *Ref* |  |  |  | - | - |  | - |
| Frequently | - | - |  | - | 0.86 | [0.35 - | 2.11] | 0.75 | - | - |  | - |
|  |  |  |  |  |  |  |  |  |  |  |  |  |
| *Baseline model: Age, sex and primary role in health system. Model fit statistics are, X² (3, N=584) = 15.40, p < .05, AIC = 408.59.* | | | | | | | | | | | | |
| *Model 1: Baseline model, personal use/non-use of social media, frequency of social media usage by types of social media platforms, organizational use/non-use of social media for population health work, and level of understanding of population health. Model fit statistics are X² (12, N=584) = 18.35, p = 0.11, AIC = 423.64.* | | | | | | | | | | | | |
| *Model 2: Backward elimination was performed on Model 1 to obtain the final model. Model fit statistics are, X² (3, N=584) = 15.40, p < .05, AIC = 408.59.* | | | | | | | | | | | | |
